# Access to artemisinin combination therapy for malaria in remote areas of Cambodia

**DOI:** 10.1186/1475-2875-7-96

**Published:** 2008-05-29

**Authors:** Shunmay Yeung, Wim Van Damme, Doung Socheat, Nicholas J White, Anne Mills

**Affiliations:** 1 Health Policy Unit, London School of Hygiene and Tropical Medicine, Keppel Street, London, WC1E 7HT, UK; 2 Wellcome Trust-Mahidol University, Oxford Tropical Medicine Research Programme, Faculty of Tropical Medicine, Mahidol University, 420/6 Rajivithi Road, Bangkok 10400, Thailand; 3 Department of Public Health, Institute of Tropical Medicine, Nationalestraat 155, B-2000 Antwerp, Belgium; 4 The National Centre for Parasitology, Entomology and Malaria Control, 372 Monivong Boulevard, Phonm Penh, Cambodia

## Abstract

**Background:**

Malaria-endemic countries are switching antimalarial drug policy to artemisinin combination therapies (ACTs) and the global community are considering the setting up of a global subsidy mechanism in order to make them accessible and affordable. However, specific interventions may be needed to reach remote at-risk communities and to ensure that they are used appropriately. This analysis documents the coverage with ACTs versus artemisinin monotherapies, and the effectiveness of malaria outreach teams (MOTs) and Village Malaria Workers (VMWs) in increasing access to appropriate diagnosis and treatment with ACTs in Cambodia, the first country to switch national antimalarial drug policy to an ACT of artesunate and mefloquine (A+M) in 2000.

**Methods:**

A cross-sectional survey was carried out in three different types of intervention area: with VMWs, MOTs and no specific interventions. Individuals with a history of fever in the last three weeks were included in the study and completed a questionnaire on their treatment seeking and drug usage behaviour. Blood was taken for a rapid diagnostic test (RDT) and data on the household socio-economic status were also obtained.

**Results:**

In areas without specific interventions, only 17% (42/251) of respondents received a biological diagnosis, 8% (17/206) of respondents who received modern drug did so from a public health facility, and only 8% of them (17/210) received A+M. Worryingly, 78% (102/131) of all artemisinin use in these areas was as a monotherapy. However, both the VMW scheme and MOT scheme significantly increased the likelihood of being seen by a trained provider (Adjusted Odds Ratios (AOR) of 148 and 4 respectively) and of receiving A+M (AORs of 2.7 and 7.7 respectively).

**Conclusion:**

The coverage rates of appropriate diagnosis and treatment of malaria were disappointingly low and the use of artemisinin monotherapy alarmingly high. This reflects the fragmented nature of Cambodia's health system in remote areas and the reliance placed by these communities on informal vendors from whom artemisinin monotherapies are widely available. However VMWs in particular are an effective means of improving access to malaria diagnosis and treatment. The VMW scheme and the social marketing of RDTS and blister-packaged artesunate and mefloquine have both been scaled up nationally. Case management in the public sector has also reportedly improved. Given recent concerns regarding the development of artemisinin drug resistance on the Thai-Cambodia border, the effectiveness of these measures in reducing the use of artemisinin monotherapy needs to be urgently re-evaluated.

## Background

Artemisinin combination therapies (ACTs) are now the official drug of choice in most malaria-endemic countries. Funds are being raised for the purchase of drugs through a global subsidy mechanism, the Affordable Medicines Facility-malaria (AMFm), with the recognition that they must be heavily subsidized in order to make them affordable to those who most need them. Malaria control programmes now face the difficult task of implementation. Whilst limiting as far as possible the inappropriate use by those who do not have malaria, there is a need to ensure that the drugs reach those who are most at-risk. This is particularly challenging because these are often poor communities in remote areas, with no access to formal health services. Depending on the local context, a number of different delivery mechanisms are possible including utilisation of the private sector and community-based strategies. However there is little experience on which to base the design of such programmes and few data to support technical and financial planning.

Cambodia provides such an experience. Although this study was conducted a few years ago, the findings are all the more relevant now in light of recent evidence that resistance may be emerging to artemisinins in the region. Cambodia was the first country to change policy nationwide to an artemisinin combination therapy of artesunate and mefloquine in 2000 [1] and in doing so also spearheaded some innovative strategies aimed at maximising the chance of successful implementation. These included specific community based interventions aimed to increase access to accurate diagnosis and treatment. In order to contribute to planning in other countries currently scaling up provision of ACTs, this analysis documents the effect of the policy change and initiatives at the level of the community in terms of coverage and adherence. The cost of these interventions is described elsewhere [2].

Cambodia lies in the Mekong delta region bordering Thailand, Lao PDR and Vietnam. The population of approximately 13.6 million is mainly rural and predominantly Khmer speaking. Following decades of turbulence and social disruption it has recently entered a period of stability and economic development.

However, malaria continues to be a major health problem particularly in the thick tropical forests, the breeding ground for the main malaria vectors, *Anopheles minimus *and *Anopheles dirus*. These areas cover between 30–56% of the land mass [3,4] and represent the most remote and inaccessible areas in Cambodia. An estimated two million people are at risk of malaria. One of the groups at highest risk, with the worst access to health care, are recent migrants from the relatively over-populated central plains, who in recent years have moved into remote forested areas that were previously dangerous and inaccessible because of conflicts, poor roads and unexploded landmines. The other groups at-risk include ethnic minority families living in thickly forested villages in Mondulkiri and Rattanakiri and a heterogenous group of "temporary forest migrants" [5].

A recent cross-sectional survey revealed (smear positive) prevalence rates of 3.0–12.3% [6]. Based on official statistics, there were 71,258 confirmed cases of malaria of which 63,739 (89%) were due to *Plasmodium falciparum*, and 492 deaths in 2006 [7]. However, as the majority of Cambodians do not seek treatment in public health facilities, these numbers significantly underestimate the true burden of disease especially in remote areas [6].

Malaria control continues to be a vertically controlled programme run by the National Centre of Entomology, Parasitology and Malaria Control (CNM) and currently receives most of its funding from the Global Fund for AIDS, TB and Malaria (GFATM).

In addition to the problem of the inaccessibility of the at-risk population, a number of other factors make the control of malaria in Cambodia particularly challenging. Like other malaria-endemic countries, most patients with fever (80–90%) use informal health providers rather than public health facilities [8-10]. These informal sector providers range from village vendors, who sell everyday goods such as cigarettes and simple drugs, to trained health workers in larger towns. The latter are a heterogeneous group composed of individuals who received training in the refugee camps or from the Khmer Rouge as well as pharmacists, nurses and doctors who are also officially employed in the public sector [11]. Diagnosis is often presumptive leading to the widespread inappropriate use of antimalarial drugs. Treatment comprises small individual packets a containing a "cocktail" of three to five different tablets including antipyretics, antibiotics, antimalarials, vitamins, antihistamines and even steroids. The number of packets, and therefore doses bought, varies according to a number of factors including what the buyer can afford and the severity of illness. There is little government control of the informal sector, which not only limits the impact of any change in treatment policy and but also has resulted in the widespread availability of sub-standard and fake drugs, and in particular sophisticated imitations of artesunate [12-14].

One of the major problems in Cambodia is multi-drug resistance, particularly on the Thai-Cambodia border [15,16]. Alarmingly, recent reports document decreasing efficacy to ACTs which, if due to artemisinin drug resistance, could have devastating consequences for global malaria control [17,18]. There are a number of factors which may have contributed to this, but the way antimalarial drugs have been deployed and used are probably contributory, particularly if artemisinin monotherapy and sub-standard drugs are prevalent.

When Cambodia changed its antimalarial drug policy to artesunate and mefloquine (A+M) in 2000 it was hailed as the first country to make a nationwide switch to an ACT [19]. The policy change was accompanied by a number of highly innovative strategies including:

▪ The local blister-packaging of the combination into three age-group packages (ages six and above), to encourage provider and patient adherence and to aid product recognition and ensure consistent drug quality. For children under the age of six years, 5 days of rectal artesunate was the recommended treatment at the time although this in currently being changed to oral A+M.

▪ The social marketing of blister-packaged artesunate and mefloquine as "Malarine^®^" in the private sector at a subsidized price.

▪ A publicity campaign to raise awareness about fake antimalarial drugs.

▪ The promotion of the use of rapid diagnostic tests (RDT), in both the public and private sector.

### Malaria outreach and Village Malaria Workers

In addition to the change in national policy, there were a few specific interventions aimed at increasing access to diagnosis and treatment, namely a malaria outreach project and the training of Village Malaria Workers (VMWs). Both of these interventions and their costs are described in more detail in a related paper [2].

The malaria outreach project was set-up and funded by Médecins Sans Frontières (MSF) as part of their programme of support in Anlong Veng district, Oddor Meanchey province. The area in the northwest of Cambodia is heavily forested and until 1999 had remained a Khmer Rouge (KR) stronghold and therefore was largely inaccessible. The collapse of KR power resulted in an influx of non-immune migrants who came in search of farmland and to collect forest products.

As a result one of the major health problems was an outbreak of malaria. Between May and August of 1999, in the health centre alone, there were over 2000 confirmed malaria cases, 400 hospitalizations and 18 deaths with malaria accounting for one third of all outpatient and two thirds of all inpatient cases [20]. At the time, the national antimalarial guidelines for uncomplicated falciparum malaria in that area was still single dose mefloquine, and for complicated malaria, quinine and tetracycline. In response to the outbreak and the known problem of multidrug resistance, MSF switched first-line treatment to an ACT of artesunate and mefloquine (A+M) and set up a malaria control project. One of the core components were malaria "outreach" teams (MOTs) which consisted of teams of two people who went out daily from the health centre to the settlements, in order to provide free diagnosis and treatment for malaria using RDTs and A+M. There were initially two teams, expanding to four teams with the aim of visiting each settlement once or twice per week depending on population movement, road conditions and information about suspected malaria outbreaks. The population of the district at the time was estimated to be 19,029 in 64 settlements (Goubert L, personal communication). This figure is continuously changing as new settlements appear and sometimes "older" ones disappear in response to local and political problems and new developments.

The first experience of using VMWs in Cambodia came from a European Commission (EC) funded community based trial for insecticide treated bed nets (ITNs) in 30 villages in Rattanakiri in the Northeast of the country in 2001 [21]. This is a remote heavily forested area, sparsely populated by ethnic minorities with low access to any kind of health service. Malaria transmission is high with cross-sectional parasite prevalence rates of between 5 and 57%. In order to address ethical concerns about having a control group without any interventions, VMWs were introduced in all villages. The VMWs were trained to perform RDTs on any villagers suspected of having malaria and to provide treatment as per the national guidelines. Diagnosis and treatment were provided free of charge and the VMWs themselves received a minimal incentive of $4 per month. They were supervised and re-supplied monthly by the provincial malaria staff. The resulting data from this passive surveillance system exposed the scale of the malaria problem and demonstrated that VMWs provided a practical means of access to diagnosis and treatment. There was a further pilot project in the Khmer speaking province of Koh Kong, after which the VMW programme has been scaled up to cover 300 villages across Cambodia [22] with support from the Global Fund.

This objective of this study is firstly to document the usage of artemisinins and other antimalarials, following the change in national policy and secondly to analyses the impact of outreach clinics and VMWs in increasing access to accurate diagnosis and treatment with ACTs.

## Methods

**Figure 1:**
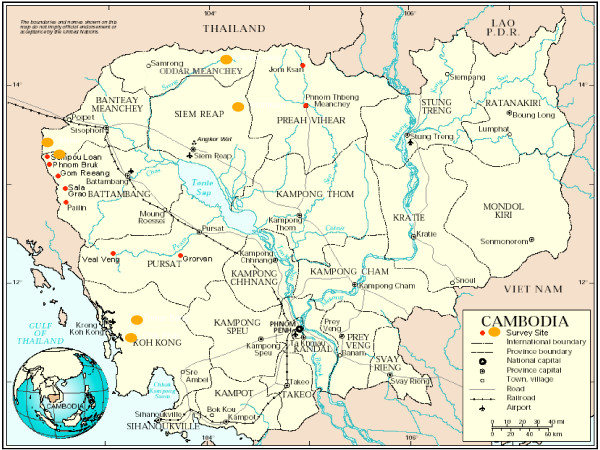
Map of study sites in Cambodia. The large yellow dots identify the areas studied in this study and the small red dots are the areas covered by the national drug usage survey carried out in 2002.

Cross-sectional household surveys were carried out in 2002, in three different types of intervention areas: with VMWs, outreach clinics and no specific interventions. Selection of the study villages was pragmatic in order to include areas with interventions, and areas without interventions which had similar characteristics in terms of risk of malaria; ecology; access to roads, health centres and markets; and the socio-economic status of the communities in terms of livelihood, poverty and level of migration. Thus the district of Anlong Veng, which was covered by the outreach programme, was selected as were villages in the Koh Kong VMW pilot project. The non-intervention areas were in the neighbouring districts of Malai, Sampalouen and Sotnikum and Thmar Bang (Figure 1). After identifying all the villages covered by the health centre, villages were excluded where no malaria cases had been seen or reported and which were deemed too dangerous (because of landmines or bandits). The remaining villages were stratified into two groups according to accessibility. Villages were then randomly selected from each stratum. Selected villages were visited one or two days before the day of the survey in order to inform and discuss the study with the local community.

### The survey

On the day of the survey, all households in the selected villages were visited and, if an adult was present, screened for inclusion. Where no adults were present, interviewers were instructed to return to the house twice if possible, before recording them as absent. The inclusion criteria included anyone in the household who in the last three weeks had either taken an antimalarial drug or who had symptoms consistent with malaria. This was defined as fever +/- headache +/- chills excluding other localizing infection e.g. bloody or profuse watery diarrhoea, or cough productive of coloured sputum as the predominant symptom. The survey instrument consisted of a household module and an individual module which had been developed and piloted following review of previous surveys [10,23,24] and pre-survey qualitative work. The household module contained questions on household socio-economic status. The individual module was filled out for each member of a household who fulfilled the inclusion criteria and included sections on the most recent and preceding episodes of fever. The questions were designed to capture some key indicators of antimalarial drug usage (Table 1). Respondents were also shown a drug identification folder as a prompt to help them recall the identity of drugs. Blood was taken by finger prick for an RDT (Paracheck^®^) to estimate the proportion of respondents who actually had *P. falciparum *malaria in the previous two weeks. This relies on one of the usually unwanted characteristics of tests based on Histidine Rich Protein 2, (HRP2) which is that it persists in the majority of treated cases for at least two weeks following effective treatment [25]. Patients who were symptomatic and RDT positive were treated as per national guidelines. An extra 200 μl of blood was also collected onto filter paper from patients who had taken mefloquine and 30 "controls" in order to measure drug levels and estimate the actual amount of mefloquine taken by comparing with population pharmacokinetic data. In the event, due to the small numbers it was not feasible to carry out this part of the study.

**Table 1:** Drug use indicators derived from questionnaire

**Objective of malaria drug policy**	**Indicator**
Increase treatment from trained provider	% of people with fever who went to a trained provider
Increase use of biological diagnosis	% of people with fever who had a biological diagnosis
Reduce delay in treatment	% of people who receive modern drugs within 48 hours of symptoms
Correct drug choice	% of first-line drug (artesunate and mefloquine) as proportion of modern drugs
Decrease use of artemisinin monotherapy	% of artemisinin monotherapy as a proportion of all artemisinin use
Adherence to ACT	% of people who take artesunate and mefloquine for at least 3 days
Reduce patient costs	Median drug cost of treatment

Ethical approval was granted by the Cambodian Ministry of Health and the research ethics committees of the London School of Hygiene and Tropical Medicine, and the Oxford Tropical Medicine Network. Informed consent was obtained from the community and individual participants.

### Data entry and analysis

Data were double-entered into EpiInfo 2000, cleaned and analysed using STATA^© ^(Version 8 and 9). The STATA survey commands were used to adjust for stratification by intervention area and to adjust for clustering between individuals in the same village. Observations were not weighted as there was no sampling within villages. Differences in proportions were tested for significance using the Pearson chi-squared statistic adjusted for the survey design. Regression models were used for multivariate analysis of variation in key outcomes using the STATA survey logistic (*svy logistic*) commands, which enable adjustments to be made for clustering and stratification.

To simplify the analysis of the treatment types, drug treatments were grouped into mutually exclusive categories that were felt to be most informative and useful for comparing with recommendations. The groups were formed hierarchically according to the presumed efficacy of the drugs (artemisinins, quinine, chloroquine, then other). For example the combination "quinine plus tetracycline" was grouped as "quinine +/- other antimalarial".

As a proxy measure of adherence, the stated duration of treatment was compared to the minimum recommended duration because of the difficulty of determining with any accuracy, the dose and timing of treatment. In addition, the adequacy of treatment duration is particularly important in determining cure and decreasing the likelihood of resistance arising to the most efficacious and commonly used antimalarials (artemisinins and quinine). For simplicity, respondents who took drugs for longer than the recommended duration were regarded as "adherent" rather than creating an extra category for prolonged duration.

In order to compare the level of poverty between households, a poverty rank was established by performing a principal component analysis (PCA) on the socio-economic indicators collected in the survey. Such indices have been accepted as reasonable proxies of socio-economic status [26].

## Results

### Household characteristics

In all, 1491 households in 23 settlements in six administrative districts were visited (Table 2). Of note is the small sample size from the VMW intervention area. This was due to political problems that necessitated the withdrawal of the survey from these villages early on in the study.

**Table 2:** Summary description of sample

	**Total**	**Intervention area**		
		
		**No intervention**	**Outreach**	**VMW**
No. of districts included	6	4	1	1
No. of villages included	23	14	7	2
Total population of included villages	10,120	8,325	1,401	394
No. of households in included villages	2,093	1,767	252	74
No. of households visited	1,491	1,185	232	74
No. of households screened i.e. with adults present (% of those visited)	1,143 (77%)	884 (75%)	198 (85%)	61 (82%)
No. of households included (as % of those screened)	290 (25%)	208 (24%)	63 (32%)	19 (31%)
No. of individuals included	361	251	88	22
Mean age in years	-	25.2	22.0	21.8
% male	-	57%	58%	55%

In total, 290 households were included in the analysis. The population was very poor, even by Cambodian standards and many were recent arrivals with 57% of households having been in-situ for less than five years. A third had to borrow rice, or money for rice, in the last year. Only 7% had access to water from a drilled well and one quarter of households were completely landless. Housing materials were of poor quality with 70% of roofs being made only of leaves or bamboo. The majority of households did not own any draft animals (85%), any means of transport (66%), or radio or television (64%). Level of educational attainment was generally low, with 42% of the adult respondents reporting having had no schooling at all.

### Individual characteristics

Altogether 361 (57% male) individuals fulfilled the inclusion criteria: 251 from non-intervention areas, 88 from MOT areas and 22 from VMW areas. The median age was 22 years, with 6% of the sample being five years or younger. Age and sex distributions did not differ significantly between different intervention areas. Of the sample, 70% (254) reported having only one episode of fever in the previous two months, where an episode was described as a period of fever separated from a previous fever by more than three days. The remainder reported having had two episodes except for two individuals who had three episodes. For the most recent episode 12% (42/361) did not receive any treatment, 56% (203/361) received one treatment, 27% (99/361) received two treatments and 5% (18/361) received three treatments.

#### Treatment with modern drugs

In non-intervention areas, modern drugs, meaning tablets or pills as opposed to traditional remedies, were taken by 85% (213/251) of the respondents for the most recent episode of fever. Surprisingly, the treatment rate was significantly lower in the VMW area with only 14 out of the 22 (63%) respondents reporting to have received modern drugs compared to 84% (74/88) in outreach areas (χ^2 ^p = 0.054). By multivariate analysis the adjusted odds ratio (AOR) of receiving modern drugs was 0.28 (95% confidence interval (CI) 0.10 – 0.80) if the respondent was from a VMW area, adjusting for age, sex, distance from health centre, poverty and clustering in the survey design (Table 3). Possible reasons for this are discussed later.

**Table 3:** Outcomes (%, number and adjusted odds ratio (AOR)) for most recent episode of fever, by intervention area*

**Outcome**	**Intervention area**				
	
	**No intervention**	**Outreach**		**VMW**	
	
	% (n)	% (n)	AOR (95% CI)	% (n)	AOR (95% CI)
Received biological diagnosis	17% (42/251)	35% (31/88)	2.4 (0.6–8.9) (p = 0.102)	**63%** **(14/22)**	**10.7 (4.7–24.3)** **(p =< 0.001)**
Received modern drugs	85% (213/251)	84% (74/88)	0.7 (0.3–1.5) (p = 0.340)	**63%** **(14/22)**	**0.28 (0.1–0.8)** **(p = 0.03)**
Received drugs from trained provider (of those who received modern drugs)	8% (17/206**)	**31%** **(23/74)**	**4.0 (1.2–13.2)** **(p = 0.023)**	**93%** **(13/14)**	**147.5 (8.5–2571)** **(p = 0.002)**
Received artesunate and mefloquine (of those who received modern drugs)	8% (17/210**)	**22%** **(16/74)**	**2.7 (1.0–7.6)** **(p = 0.053)**	**64%** **(9/14)**	**7.7 (1.8–28.2)** **(p = 0.007)**
Paid > $1 for treatment (of those who received modern drugs)	52% (110/210**)	46% (34/74)	1.2 (0.6–2.1) (p = 0.525)	29% (4/14)	0.55 (0.2–1.9) (p = 0.107)

*Adjusted for sex, age (< 6 and 6–14 years), distance from health centre (> 2 hours by motorcycle), poverty rank (poorest 40% and richest 20%) and survey design. Results in **bold **highlight variables that significantly affect the AOR.

**Missing data therefore does not add up to 213

#### Timing of treatment

Overall, the median time to modern treatment from the onset of symptoms was two days. "Delay in treatment" was defined as time to treatment of more than two days. In non-intervention areas, the delay in treatment was 37% (107/289), compared to 25% (19/75) in outreach areas and 57% (8/14) in VMW areas (χ^2 ^p = 0.026). When adjusted for clustering, this difference was no longer significant (p = 0.1536). This suggests that clustering may have contributed to the significance of the result and that the sample may have been underpowered to detect the difference between interventions. By multivariate analysis the only factor to correlate with delay in treatment was age five years or under, for whom there was less likelihood of delay (AOR 0.58, 95% CI 0.35–0.98, p = 0.044).

#### Source of treatment

In non-intervention areas, the single most common source (54%, 121/224) of initial treatment was a local village vendor. This was followed by private health workers, either at the providers' place (24%, 54/224), or at the patient's home (6%, 12/224)). Village vendors were also the most popular first source of treatment in areas with MOTs (63%, 48/76), with only 17% (13/76) of respondents first using the outreach service. However this was significantly different in areas with VMWs, where 71% (12/17) of respondents did first visit the VMW (Table 4).

**Table 4:** First source of treatment by intervention area (n)

**First source of treatment**	**Intervention area %**		
	
	**None n = 317**	**Outreach n = 76**	**VMW n = 17**
Village vendor	54%	63%	12%
Public health facility	6%	4%	0
Went to private practitioner	24%	7%	6%
Private practitioner came to home	6%	1%	0
VMW	1%	0	71%
Outreach	0.5%	17%	6%
Traditional healer^1^	3%	0	0
Other^2^	6%	8%	6%

^1^The number of "Traditional" treatments is probably underestimated. Patients often use local remedies early on in an illness, at the same time as modern medicines. These included local plants, rubbing with coins or cupping.

^2^"Other" included the military, de-mining organisations and forest rangers.

Providers were grouped into trained (public, VMW, outreach) and "informal" (village vendors, private health worker, other). In all, only 8% (17/206) of cases in non-intervention areas who received modern drugs, received them from a trained provider. This was much higher in both outreach villages (31%, 23/74) and particularly VMW villages (93%, 13/14) (p < 0.001) (Table 3). Children under the age of 14 were three times more likely to be taken to see a trained provider, but neither distance from the nearest health centre, sex, or level of poverty made any difference.

#### Diagnosis

Overall rates of biological diagnosis were very low. Only 17% of 251 individuals in non-intervention areas reported having had a biological diagnosis for their most recent episode of illness. This was significantly higher in the areas with VMWs and outreach, at 63% (14/22) in the former and 35% (31/88) (p = 0.009) in the latter (Table 3). By univariate analysis the odds ratio of having a test were 8.5 (95% CI 3.3–21.4) if the respondent was from a VMW area and 2.6 (95% CI 1.5–4.5) if they were from an outreach area. Adjusting for the same factors as described previously, the odds ratio of having a test was increased 11-fold in VMW areas and two-fold in outreach areas but adjustment for clustering reduced the level of significance, so that only the former reached significance (Table 3). This reflects the variance between villages in non-intervention areas with 2% of respondents from villages in Chik Phat reporting having had a biological diagnosis compared to 46% in Malai. Children aged six to 12 years were three times more likely to have a test (95% CI 1.1–7.8, p = 0.035), than other age groups. Sex, distance from health centre and level of poverty did not have any effect.

The difference seen in intervention areas is mainly explained by the fact that only 18% of the interactions resulted in a biological diagnosis in the private sector, the most popular source of treatment in non-intervention areas. This compares to all consultations by VMWs and outreach workers resulting in a biological test. The type of test performed also varied significantly by intervention area so that VMWs and outreach workers reportedly always used RDTs. In contrast, only 15% (6/41) of tests at private health facilities and 69% (11/16) of tests at public health facilities were by RDT (Table 5).

**Table 5:** Biological diagnosis, by type of provider

**Type of provider**	No. of contacts (n = 452)*	**No. (%) of contacts resulting in biological diagnosis (A)**	**No. of RDTs (as % of A)**	**No. of tests reported positive (as % of A)**
Village vendor	232	3% (8)	38% (3)	50% (4)
Went to private practitioner	98	42% (40)	15% (6)	88% (36)
Public health facility	31	52% (16)	69% (11)	81% (13)
Private practitioner came to home	24	17% (4)	3% (1)	75% (3)
VMW	18	94% (17)	100% (17)	65% (11)
Outreach	23	96% (21)	100% (22)	77% (17)
Other	26	15% (4)	75% (3)	75% (3)

*Number of contacts is greater than number of individuals because some individuals had more than one contact

The reported rate of positive tests was generally high, but significantly lower if performed by RDT (67%) compared to microscopy only (88%) (χ^2 ^p = 0.009). This difference is reflected in the decreased likelihood of having a positive test if performed by a VMW (65%, 11/17) or an outreach worker (75%, 3/4) compared to going to a private facility or public health facility (Table 5). The relationship between reported test results and treatment received is explored below.

#### Type of antimalarial drugs

Respondents reported receiving altogether 464 treatments in the previous two months. As explained, treatment usually consisted of little plastic bags containing a "cocktail" mixture of different drugs. These contained a mean of 2.6 different tablets with 23% containing four or more tablets. Of these treatments 63% (296) were known to contain an antimalarial, 13% possibly contained an antimalarial ("unknown") and 23% did not.

Within these treatments there were at least 28 different combinations of antimalarials of which 15 contained an artemisinin derivative. The type of treatment obtained varied according to the provider. The most popular treatments from village vendors contained no antimalarials (30%, 56/188), chloroquine (26%, 49/188) or an artemisinin monotherapy (22%, 41/188), followed by treatments containing quinine (12%, 22/188). For treatments from private practitioners only 12% (11/95) contained A+M, 34% (32/95) contained an artemisinin monotherapy and 25% could not identified (24/95). The remaining contained chloroquine (11%, 10/95) or quinine (13%, 12/95). From public health facilities, 29% (8/28) of treatments could not be identified and A+M and quinine (with or without tetracycline) made up 14% (4/28) each. Artesunate monotherapy and choloroquine each accounted for 11% (3/28) of reported treatments. From both VMWs and MOTs, three-quarters of treatments received were identified as A+M (13/17 and 16/21 respectively). The remainder being made up of no antimalarials or unidentifiable treatments.

Comparison of the reported results of diagnostic tests with the reported treatments received, suggest that VMWs and MOT workers generally prescribed according to the blood test results. Eleven out of seventeen (65%) respondents who had RDT tests performed by VMWs, reported positive results, all of whom received A+M. Two of six respondents with negative tests also reported receiving A+M. For respondents who had tests performed by MOTs, 16 out of 21 (76%) reported positive tests of whom 15 received A+M and one reported receiving no treatment. Conversely, one of five respondents with a negative test reported receiving A+M. Of the 16 respondents who had diagnostic test in public health facilities, 13 (81%) reported positive results of whom only three reported receiving A+M. The other received quinine (3), chloroquine (3), other or unknown (4) and none (1). For tests performed by private practitioners 89% (39/44) were reported positive of whom only 5/39 (13%) reported receiving A+M. This compares to 5/51 (10%) with no tests and 1/5 (20%) for reportedly negative tests.

Analysis according to intervention area showed that in non-intervention areas, the most commonly received mixtures were ones containing artesunate *without *mefloquine, which accounted for 40% (102/257) of all treatments containing antimalarials (Table 6). These treatments can be effectively considered as artemisinin "monotherapy" because even if they contained other antimalarial drugs, the latter were generally either ineffective against *P. falciparum *malaria (e.g. chloroquine) or were taken for insufficient duration (e.g. quinine). In non-intervention areas this accounted for 78% (102/131) of all artemisinin derivative use whereas in outreach and VMW areas this was 36% (10/28) and 7% (1/14) respectively.

**Table 6:** Type of antimalarial therapy received, by intervention area

**Antimalarial therapy received (%)**	**Intervention area % (n)**		
	
	**None n = 224**	**Outreach n = 78**	**VMW n = 21**
Artesunate + mefloquine	11% (29)	23% (18)	52% (13)
Artemisinin derivative +/- other antimalarial	40% (102)	13% (10)	5% (1)
Quinine +/- other antimalarial	9% (22)	12% (9)	0
Chloroquine +/- other antimalarial	24% (62)	38% (30)	0
Unknown	16% (42)	14% (11)	33% (7)

P < 0.001

In order to compare coverage with the first-line ACT, artesunate and mefloquine, information on antimalarial treatments taken for the most recent episode were compared (Table 3). In non-intervention areas only 8% of patients received artesunate and mefloquine compared to 22% and 64% in the outreach and VMW areas respectively (p < 0.001). The adjusted odds ratio of receiving A+M was 2.7-fold higher in outreach areas and 7.7-fold higher in VMW areas. No other factor was significantly associated with a change in likelihood of receiving A+M.

#### Adherence

Most antimalarial drugs were taken for a median of two to three days with a range of one to 14 days. As shown in Table 7, adherence was better to the three-day regime of A+M than to the three-day regime of chloroquine (77% (34/44), versus 35% 22/63)). However when artesunate was not taken as part of pre-packaged artesunate and mefloquine, adherence was poor. If it was taken as part of a cocktail of drugs containing another antimalarial, 13% (4/31) of respondents reported taking it for the required 7 days. However if it was taken alone, without another antimalarial drug, adherence was higher at 28% (8/29), reflecting the occasional practice where patients would buy a blister-pack of 12 artesunate tablets rather than individual packages of mixed drugs. Overall adherence to 7-day treatment of an artemisinin without mefloquine was 20% (12/60). Adherence was even poorer with the less palatable quinine-based regimes with none of those taking quinine and tetracycline completing the recommended seven days.

**Table 7:** Adherence and cost (in US$ (2005)) for most recent treatment

**Regime (Recommended duration)**	**% achieving recommended durations (n)**	**Median cost in US$ (range)**
Artesunate + Mefloquine (3 days)	77% (34/44)	0.77 (0 – 12.82)
Artemisinin derivative + other antimalarial (7 days)	13% (4/31)	1.67 (0.22 – 38.90)
Artemisinin alone* (7 days)	28% (8/29)	2.05 (0.38 – 26.70)
Quinine + tetracycline (+/- other antimalarial) (7 days)	0% (0/13)	1.11 (0.35 – 17.3)
Quinine (+/- other antimalarial) (7 days)	13% (3/24)	0.66 (0 – 11.10)
Chloroquine (+/- other antimalarial) (3 days)	35% (22/63)	0.67 (0.11–11.11)
Unknown	-	3.59 (0 – 41.03)

* Based on studies of artesunate monotherapy [41-43].

By intervention area, the likelihood of being adherent to an antimalarial regime reflected the likelihood of receiving A+M so that in non-intervention areas adherence was 37% (80/218), in outreach areas 44% (34/78) and in VMW areas was 90% (17/19). By univariate analysis the odds (OR) of being adherent was 15-fold greater in VMW areas (95% CI 3.3–65.1) but not significantly greater in outreach areas. By multivariate analysis adherence was not affected by age, sex, distance from closest public health facility, schooling or level of poverty (data not shown).

#### Community effectiveness

In order to estimate community effectiveness, the probability that a patient with malaria would receive an effective course of antimalarials was estimated from the product of the probability of receiving an efficacious antimalarial, defined as either artesunate with mefloquine or quinine, and the probability of being adherent. Adherence was based on the behaviour of the aggregate sample because of the small sample size and was taken as 77% (34/44) for artesunate with mefloquine and 8% (3/37) for quinine, with or without tetracycline. This resulted in a community effectiveness of 9% in non-intervention areas, 19% in outreach areas and 40% in the VMW area.

#### Rapid diagnostic test results of the study

Overall 355 study RDTs were performed at the time of interview, of which 28% were positive. There was no significant difference in positivity rate between intervention areas and non-intervention areas. There was however considerable heterogeneity within the non-intervention group such that in Malai only one out of 44 people (2%) tested positive compared to 40 out of 91 (44%) in Chik Phat. There was no significant difference in the result if the analysis was limited to the 215 who began treatment recently (in the last three weeks) and who therefore might have been expected to still be RDT positive had they been parasitaemic before starting treatment. By multivariate analysis (Table 8), in the model adjusted for study design, the only factor significantly associated with an increased likelihood of having a positive RDT on the day of the study was being a child less than five years of age (AOR 2.5, CI 1.44 – 4.21, p < 0.001).

**Table 8:** Likelihood of positive study RDT**

**Variable**	AOR (95% CI)*	**p**
Outreach area	1.62 (0.88–3.01)	0.12
VMW area	1.06 (0.32–3.49)	0.92
Did not receive A+M in last 2 months	1.95 (0.97 – 3.91)	0.06
Did not receive any antimalarial received in last 2 months	1.04 (0.54–2.02)	0.90
Female	1.26 (0.77–2.07)	0.36
**Child 6–14 years**	**2.46 (1.44–4.21)**	**0.00**
Child < 6 years	0.64 (0.18–2.34)	0.50
**Far (> 2 hours by motorcycle)**	**0.45 (0.26–0.79)**	**0.01**
Poorest 40%	1.20 (0.67–2.15)	0.54
Richest 20%	1.14 (0.57–2.32)	0.71

Number of observations = 354

* Adjusted for study design. Results in **bold **highlight variables that significantly affect the AOR.

#### Cost to households of malaria

For each treatment sought, respondents were asked the cost of consultation, diagnosis, drugs, transport (for the patient and any companions), and "other" costs including food. In the event, few respondents were able to report disaggregated costs for consultation, diagnosis and therefore the costs are presented as reported drug costs, travel costs and total costs are shown (Table 9). The median direct costs to households for an episode of malaria was $1.28 in a non-intervention with a maximum reported cost of $55. This compared with a median cost of $0.90 (range 0–$33.70) in MOT areas and $0 (maximum $5.13) in the VMW areas. This difference in cost between intervention areas was mainly explained by the fact that the cost of drugs from outreach teams and VMWs was either very low or completely free, whereas drugs from village vendors and private providers, the most popular sources of treatment in non-intervention areas, cost a median of $0.77 and $2.95 respectively (Table 10).

**Table 9:** Costs for most recent treatment episode by intervention area

**Intervention (n)**		**Cost (US$)**		
		
		**Drugs**	**Travel**	**Total including other costs (e.g. food)**
None (205)	Mean (s.d.)*	3.24 (6.23)	0.38 (1.18)	4.31 (8.07)
	Median (range)	0.88 (0 – 41.0)	0.00 (0–11.1)	1.28 (0 – 54.9)
Outreach (71)	Mean (s.d.)	2.01 (3.22)	0.26 (0.89)	2.92 (5.32)
	Median (range)	0.77 (0 – 17.9)	0.00 (0–6.41)	0.90 (0 – 33.7)
VMW (16)	Mean (s.d.)	0.52 (1.28)	0.00	0.68 (1.37)
	Median (range)	0.00 (0–5.13)	0.00 (0–0)	0.00 (0–5.13)

*s.d = standard deviation

**Table 10:** Cost (US$) of drugs (for most recent treatment episode) by provider type

**Provider**	**n**	**Median (Range)**	**Mean (s.d.)**
Village vendor	151	0.77 (0 – 12.82)	1.28 (1.86)
Went to private health worker	65	2.95 (0 – 41.03)	7.35 (9.56)
Public	17	0.95 (0 – 9.23)	2.41 (2.83)
Private health worker came to home	15	6.44 (0.44–15.38)	5.73 (4.23)
VMW	12	0 (0 – 1.78)	0.23 (0.54)
Outreach	18	0.64 (0 – 1.28)	0.51 (0.41)
Other	13	0 (0 – 1.54)	0.30 (0 – 0.57)
Total	291	0.77 (0 – 41.03)	2.79 (5.51)

#### Cost to households of drugs

Overall, households spent a median of $0.77 for A+M compared to $1.67 for treatments containing an artemisinin derivative and another (non-mefloquine) antimalarial, and $2.05 for treatments containing only an artemisinin derivative. Treatments that did not contain any antimalarial cost $0.38 and the most common non-artemisinin antimalarial treatments cost a median of $0.67 (for both chloroquine and/or tetracycline and quinine without tetracycline) (Table 7). There was no difference between intervention areas in the likelihood of paying more than $1 for treatment (Table 3). By multivariate analysis, only the level of poverty affected the likelihood of paying more than $1 for drugs with those in the poorest 40% being less likely than those in the middle 40% AOR 0.38, 95% CI 0.21–0.68).

## Discussion

This study sought to document the impact of the change in malaria treatment policy to artemisinin combination treatments (ACTs) and the impact of different delivery strategies in Cambodia, the first country to make a nationwide change in policy to ACTs. It differed from other treatment seeking behaviour studies in also trying to estimate the proportion of fever cases that were due to malaria through the use of RDTs.

The main limitation of this survey is that the sample size was relatively small. This was largely due to the difficulties related to carrying out fieldwork in the remote jungle areas where malaria occurs. Many communities were located far from roads and were only accessible by foot or canoe. The study was conducted in the rainy season, when malaria is most prevalent but road conditions are at their worst. In addition, in some areas there was a risk of unexploded landmines which further limited access. The sample size from the VMW intervention area was particularly small because for political reasons, the study had to be withdrawn early from the area. The main consequence of the small sample size is that some of the apparent differences between interventions and non-interventions areas failed to reach statistical significance, after adjusting for study design. However despite this limitation, significant differences between interventions areas were still found.

Other limitations include the fact that this was an observational study undertaken after interventions had already been implemented, and the sampling design which purposively selected areas known to be malaria endemic rather than a completely random selection. This was done in order to limit the time and resources that would be wasted in visiting villages with little malaria. However, as discussed below, comparison with a survey conducted the following year showed that the results were not dissimilar [27].

As in other similar studies there were also potential biases inherent in this kind of study. These include the inaccuracies in reported drug usage and results of diagnostic tests. Recall bias with a tendency to recall positive rather than negative test results may explain the surprisingly high number of positive tests. To assist in validating the responses, where possible, respondents' descriptions were compared to available records and a drug identification board was used. There was also a potential bias in excluding households in which an adult was not present at the time of the study. In order to mitigate against this, communities were forewarned of the day of the study and the study itself was timed just after the planting season, when village occupancy was close to a maximum.

The first major finding in this study were that coverage of ACTs was low and that there was widespread use of artemisinin monotherapy, constituting 78% of all artemisinin containing treatments in non-intervention areas. The other major finding was that significant improvements could be made with specific interventions to improve access to reliable diagnosis, and free drugs.

The findings of low ACT coverage in this study were later confirmed in a larger national antimalarial drug usage survey the following year in nine districts in which 1277 household respondents in 36 villages participated. An even higher proportion (92%) of treatments containing an artemisinin derivative were taken without mefloquine if they were not blister-packaged with mefloquine and only 11% of adults and 2% of 116 children under five years received treatment according to national guidelines [27].

The main reason for the low coverage rate with A+M in Cambodia was that most treatments were obtained in the private sector and, as previously reported [28], treatment in the private sector is often inappropriate. In the absence of any interventions, only 8% of respondents sought treatment from a public health facility for the most recent episode of a malaria-like illness, with the most popular first source of treatment (56%) being untrained village providers. These findings are similar to rates found in other settings with poorly functioning health systems [29-32] and confirm previous reports in Cambodia. In other studies in Ko Kong, only 2 to 4% [33,34] of patients first sought treatment for malaria in the formal sector. In other areas rates of 7 and 26% have been described [24,27].

These findings are particularly significant in view of recent reports suggesting that artemisinin drug resistance may have emerged in Cambodia. Since this study, case management in the public sector has improved with higher rates of biological diagnosis and treatment with ACTs [22]. In addition the VMW scheme and social marketing blister-packaged artesunate and mefloquine (Malarine ^®^) and RDTs have been scaled up. However, artemisinin monotherapies continue to be available now despite the World Health Organization ban [35] and action to further limit their availability and use is urgently needed. Interventions are needed to improve the quality of service provided by private providers including through training [36-38] and incentives, including the subsidy of appropriate co-formulated ACTs. Urgent consideration should be given to Cambodia as a priority country for the roll-out of the AMFm. The findings in this study of low rates of diagnosis, high rates of usage for modern drugs (85%) and the predominant use of "cocktail" mixtures of drugs suggests that behaviour change communication needs to emphasize diagnosis, and the purchase and use of complete packages appropriate ACTs.

Clearly, priority should also be given to the delivery of affordable good quality treatment through the public sector. The findings in this study suggest that VMWs in particular, appeared to be effective means of doing this. The likelihood of receiving a biological diagnosis was increased 11-fold and the likelihood of receiving A+M was increased eight-fold. In addition, the use of artemisinin derivative monotherapy was much lower than in non-intervention areas. Surprisingly however, in the VMW area a higher proportion of patients reported remaining untreated despite having had malaria-like symptoms and waited for longer before seeking treatment. This could be for a number of reasons. The presence of a VMW in the village may have allowed malaria patients to risk waiting for longer to see how symptoms evolved before seeking treatment especially if they had previous experience of not receiving any treatment following a negative diagnosis. Alternatively the delay may have been due a difficulty in finding the VMW if they were in the field or elsewhere. There may have also been personal reasons for the villagers not to consult the VMW although VMWs were selected through a community consensus.

Coverage was lower than anticipated with the outreach programme. One of the main reasons given by villagers was that because the service was intermittent, they would often end up seeking treatment from the private sector rather than waiting for the next visit by the MOT. Although coverage was lower than in villages with VMWs, it must be stressed that the decision to implement MOTs rather than VMWs because of the unique requirements of the Anlong Veng setting. Much of the population had arrived recently from disparate locations with little connection between them and individual households were struggling to survive, building themselves crude shelters and clearing land. It was therefore felt that there was little capacity or inclination for community volunteers. It may also be that there was a difference in the philosophy of the organizations involved which meant that different systems were favoured, with MSF being primarily an emergency relief organization where medical treatment is usually delivered by qualified providers, compared to the EC-malaria project which was more concerned with finding a means of increasing access that could be sustained and applied elsewhere in Cambodia.

Reassuringly there appeared to be some recognition that young children were particularly at risk of malaria, as they were significantly more likely to receive modern treatment within two days of symptoms and were three times more likely to be seen by a trained provider sector and to receive a biological diagnosis than older children and adults. However, they were no more likely to receive the recommended A+M than older age groups.

Data on drug costs suggested that households pay $0.5 to $2.60 with a median of $0.77 for antimalarial drugs and this was in fact the median costs paid for blister-packaged A+M. The median cost of a course of treatment that contained only an artemisinin drug on its own *without *another antimalarial was $2.05 (range $0.90–2.69). Interestingly this was more than treatments which contained an artemisinin drug and also another non-mefloquine drug such as chloroquine $1.67 (range $0.77–5.13). This was because artesunate tablets, the most popular form of artemisinin derivative, were generally sold either in whole blister packets containing 12 artesunate tablets at a usual cost of around $2 per packet, or as a single tablet packaged within a "cocktail package" containing a number of other cheaper drugs such as paracetemol and chloroquine to constitute a single dose of treatment at a cost of about $0.25–0.50 per packet. For the latter, the number of doses of treatment bought would depend on a number of factors including how much the patient could afford and how ill they were. The only variable with a significant correlation to the amount of money paid for treatment was poverty rank. This confirms previous findings of the relationship between poverty and treatment expenditure [39] and adds weight to the argument that ACTs will need to be provided either free or almost free if the biologically vulnerable (who are usually the most economically vulnerable) are to be reached [40].

Reassuringly, the cost of treatment was less from trained providers than from the informal sector. Treatment from the VMWs was supposed to be provided free and the study suggested that usually this was the case, although there was evidence that small payments were made occasionally (maximum $0.54). The median cost of treatment from an outreach worker was reported to be $0.64 but ranged up to $5.10. This is of concern as patients were supposed to be charged only up to a maximum of $0.75 depending on their means (as assessed informally by the outreach worker) and it was said that the majority of patients received their treatment for free. In contrast, treatment from an informal village vendor cost a median of $0.77 and from a private health worker $2.95 if the patient went to the health worker and $6.44 if the health worker came to the patient's house.

## Conclusion

This study has shown that a key challenge in changing malaria treatment policy to ACTs is ensuring adequate access to accurate diagnosis and ACTs in poor rural areas where communities have limited access to any kind of health care. Coverage was low in areas without specific interventions to increase access and the use of artemisinin monotherapy was alarmingly high. This is particularly significant in view of recent reports of possible artemisinin drug resistance arising on the Thai-Cambodia border [17]. Despite the small sample size, the study suggests that the provision of free diagnosis and treatment through trained VMWs is an effective means of increasing coverage in certain settings. Although the use of MOTs did not appear to increase coverage as much as VMWs, they were deployed in a very difficult area for malaria control with a fluid population of new migrants.

The Village Malaria Worker scheme and social marketing of RDTS and blister-packaged artesunate and mefloquine have been scaled up nationally. Case management in the public sector has improved. Given recent concerns regarding the development of artemisinin drug resistance on the Thai-Cambodia border, the effectiveness of these measures in reducing the inappropriate use of artemisinin monotherapy needs to be urgently re-evaluated.

## Abbreviations

A+M: Artesunate and Mefloquine; AOR: Adjusted Odds Ratio; ACT: Artemisinin Combination  Therapy; CI: Confidence Interval; CNM: National Malaria Centre (Cambodia); CPE: Cambodian  Pharmaceutical Enterprise; GFATM Global Fund for AIDS: TB and malaria; EC: European  Comission; HRP2: Histidine Rich Protein 2; ITN: Insecticide Treated Nets; KR: Khmer Rouge;  MOT: Malaria Outreach Team; MSF: Mèdecins Sans Frontières; NGO: Non-governmental  Organization; PCA Principal Component Analysis; PSI: Population Services International; RDT:  Rapid Diagnostic Test; SD: Standard Deviation; SP: Sulphadoxine-Pyrimethamine; VMW: Village  Malaria Worker; WHO: World Health Organization.

## Authors' contributions

SY designed the study, carried out the data collection and analysis, and drafted the paper, WVD facilitated the collection of data and made substantial contributions to analysis of data and writing of the paper, DS participated in the study design and co-ordination of fieldwork, NJW conceived of the study and participated in writing the paper, AM participated in the study design and helped to draft the paper. All authors read and approved the final manuscript.
